# Disentangling the Complexity of Nutrition, Frailty and Gut Microbial Pathways during Aging: A Focus on Hippuric Acid

**DOI:** 10.3390/nu15051138

**Published:** 2023-02-24

**Authors:** Andrea Ticinesi, Angela Guerra, Antonio Nouvenne, Tiziana Meschi, Stefania Maggi

**Affiliations:** 1 Department of Medicine and Surgery, University of Parma, Via Antonio Gramsci 14, 43126 Parma, Italy; 2 Geriatric-Rehabilitation Department, Azienda Ospedaliero-Universitaria di Parma, Via Antonio Gramsci 14, 43126 Parma, Italy; 3 Microbiome Research Hub, University of Parma, Via delle Scienze 7, 43124 Parma, Italy; 4 National Research Council, Neuroscience Institute, Via Giustiniani 2, 35128 Padova, Italy

**Keywords:** polyphenol, gut microbiota, frailty, sarcopenia, cognition, benzoic acid, diet

## Abstract

Hippuric acid (HA) is a metabolite resulting from the hepatic glycine conjugation of benzoic acid (BA) or from the gut bacterial metabolism of phenylalanine. BA is generally produced by gut microbial metabolic pathways after the ingestion of foods of vegetal origin rich in polyphenolic compounds, namely, chlorogenic acids or epicatechins. It can also be present in foods, either naturally or artificially added as a preservative. The plasma and urine HA levels have been used in nutritional research for estimating the habitual fruit and vegetable intake, especially in children and in patients with metabolic diseases. HA has also been proposed as a biomarker of aging, since its levels in the plasma and urine can be influenced by the presence of several age-related conditions, including frailty, sarcopenia and cognitive impairment. Subjects with physical frailty generally exhibit reduced plasma and urine levels of HA, despite the fact that HA excretion tends to increase with aging. Conversely, subjects with chronic kidney disease exhibit reduced HA clearance, with HA retention that may exert toxic effects on the circulation, brain and kidneys. With regard to older patients with frailty and multimorbidity, interpreting the HA levels in the plasma and urine may result particularly challenging because HA is at the crossroads between diet, gut microbiota, liver and kidney function. Although these considerations may not make HA the ideal biomarker of aging trajectories, the study of its metabolism and clearance in older subjects may provide valuable information for disentangling the complex interaction between diet, gut microbiota, frailty and multimorbidity.

## 1. Introduction

The burden of frailty and disability in our aging society is amplifying the demand for reliable biomarkers that can help clinicians and researchers to better identify the aging trajectory of older persons [1,2]. Recently, hippuric acid has been proposed as one of these markers, because its metabolism is strongly dependent on the exposure to environmental factors, such as diet, and intrinsic host factors, such as the gut microbiota, that play an important role in the pathophysiology of frailty [3].

**Figure 1:**
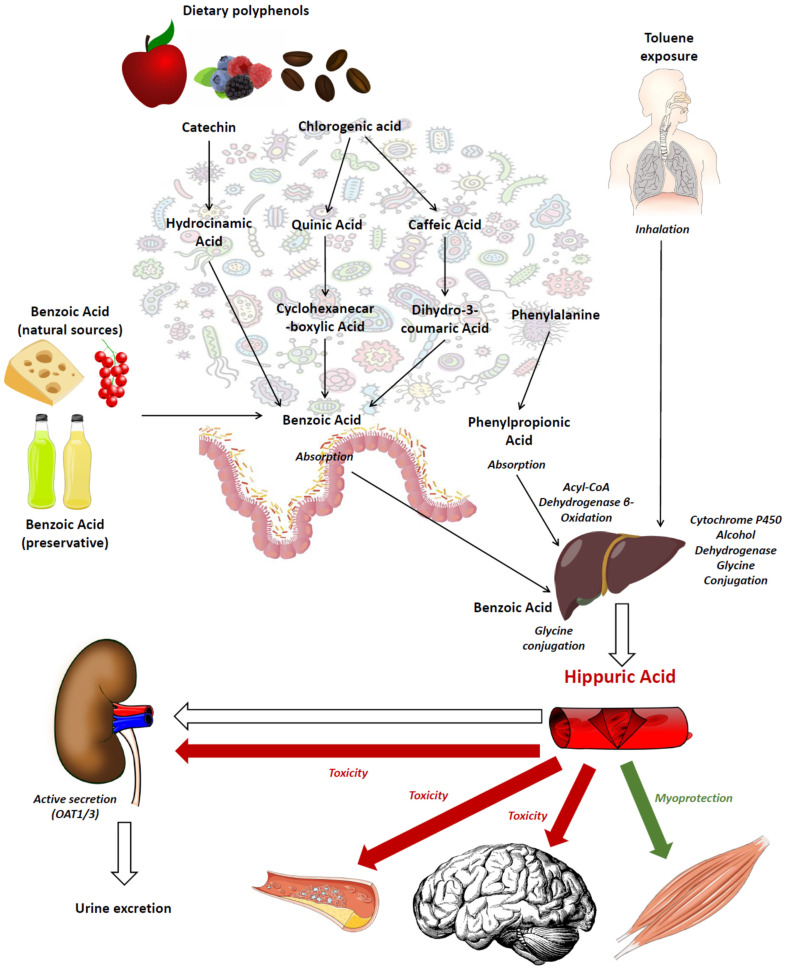
Schematic representation of pathways leading to hippuric acid synthesis in human beings, putative actions of this compound on the target organs and mechanisms of excretion.

Hippuric acid is the glycine conjugate of benzoic acid. In human beings, it was first studied in the fields of occupational medicine and toxicology because it represents the final metabolite of toluene detoxification metabolic pathways [4]. After toluene exposure, the liver cytochrome P450 enzymes produce benzyl alcohol, which is then transformed into benzoic acid by alcohol dehydrogenase. Benzoic acid is, finally, subjected to conjugation with glycine to form hippuric acid, which is then excreted into the urine (Figure 1) [4]. For this reason, urinary hippuric acid excretion is even today used to monitor workers’ exposure to toluene in high-risk occupations [5,6].

In the last decade, human metagenomics and bacterial metabolism studies have contributed to boosting our knowledge about the role of the gut microbiome in human health [2,7]. Several compounds contained in foods undergo a relevant metabolic transformation by the gut bacteria resulting in the production of bioactive compounds with important physiological functions for the host or with clinical significance as biomarkers of physiological processes [8,9]. Hippuric acid is among these compounds, representing one of the final microbial metabolites of dietary polyphenol biotransformation [9,10]. Additionally, hippuric acid may originate from the bacterial metabolism of phenylalanine [11].

The possible pathways leading to hippuric acid synthesis in human beings are summarized in Figure 1. One pathway involves epicatechins, a category of compounds belonging to the flavonoid polyphenol subclass, which are frequently found in tea, pomes, berries, broad beans and barley, among others; another pathway involves chlorogenic acids, a family of polyphenol esters conjugates with quinic and caffeic acids, found in coffee beans, berries and other fruits [10,12,13]. Both of these pathways converge in the gut microbiome-mediated synthesis of benzoic acid, which is absorbed into the circulation and transformed into hippuric acid after glycine conjugation in the liver or, to a lesser extent, in the kidney [14,15]. Benzoic acid can also be present naturally in foods, especially in berries, milk and dairy products, or artificially as a preservative, especially in beverages and other industrial foods [16]. An alternative pathway, recently discovered, involves the amino acid phenylalanine resulting from gut bacterial metabolism or food digestion, which is converted into phenylpropionic acid by the bacterial metabolism, absorbed into the circulation and then subjected to acyl-Coenzyme-A (acyl-CoA) dehydrogenase β-oxidation in the liver to form hippuric acid [11].

In the blood, hippuric acid is approximately 30–35% bound to albumin [17]. Its pathophysiological functions in the human body are, however, not fully understood to date [18]. Some in vitro studies suggest that it may exhibit myoprotective properties when incubated with skeletal muscle cells, stimulating glucose metabolism, preserving mitochondrial functions and promoting protein synthesis [19,20].

Conversely, hippuric acid inhibits Organic Anion Transporter (OAT) 3 function in neurons, favoring the toxic action of other compounds, including indoxyl sulfate [21]. At high concentrations, hippuric acid may also exert toxic effects on renal tubular cells by disrupting the redox balance through the downregulation of the Nuclear Factor Erythroid-2 (NRF2) transcription factor, which is responsible for the expression of antioxidant enzymes [22], and on endothelial cells, where it can induce massive mitochondrial reactive oxygen species (ROS) production [23] and synthesis of miR-92a, a mediator involved in atherosclerosis related to chronic kidney disease (CKD) [24]. Hippuric acid is then excreted by the kidneys through active secretion mediated by OAT1 and OAT3 [25].

Despite its potentially toxic effects, the plasma and urine levels of hippuric acid have been generally considered a marker of good health and a healthy lifestyle. In fact, hippuric acid is mostly derived from polyphenols and polyphenol-derived compounds, whose nutritional intake has been associated with several positive health effects, especially in aging [26]. In particular, the polyphenol intake has been associated with longevity, extended health span and protection against cognitive decline [27,28,29].

In this context, disentangling the real significance of plasma or urine hippuric acid levels in older individuals may result particularly challenging. As people age, in fact, changes in dietary habits, alterations in liver and kidney function, chronic illnesses and gut microbiota dysbiosis may significantly disrupt hippuric acid synthesis and metabolism [18,30,31]. Therefore, the objective of this narrative review is to critically assess the multiple factors contributing to the variability of the plasma and urine hippuric acid levels in older individuals in order to shed light on the complex interplay between nutrition, microbiota and frailty in this population.

## 2. Urinary Hippuric Acid as a Nutritional Marker

Several experimental studies have shown that both serum and urinary hippuric acid levels increase after the ingestion of foods or beverages with a high polyphenol load [32,33,34,35,36]. For example, cranberry juice consumption is associated with an increase in serum hippuric acid levels and urinary 24 h hippuric acid excretion in healthy young adults [32,33]. According to these studies, the serum hippuric acid levels were also associated with beneficial metabolic effects of polyphenol metabolites, such as the improvement of insulin secretion, the reduction of fasting plasma glucose and a favorable alteration of the post-prandial serum lipid load [35,36].

In this context, the 24 h urinary hippuric acid excretion has been proposed and validated as a marker of fruit and vegetable (FAV) intake, in view of the fact that polyphenols are mainly present in these categories of foods [37]. A high FAV intake is associated with a reduced risk of a large number of chronic diseases, including diabetes and obesity, whose burden is dramatically rising worldwide [38]. Traditional methods of assessment of the FAV intake, such as the use of food frequency questionnaires or dietary diaries, suffer from several limitations, including recall bias [39,40]. Therefore, the availability of laboratory markers of FAV intake can be considered extremely important for both clinical and research purposes, especially for those individuals who generally have the lowest FAV intakes, such as children and obese subjects [38].

The twenty-four-hour urinary hippuric acid excretion showed a fairly good correlation with FAV intake, estimated from 3-day weighted dietary records, in 287 healthy adolescents residing in Germany (unadjusted *r* = 0.64) [41]. Similarly, hippuric acid excretion was correlated with the baseline intake of FAV, detected through two 24 h dietary recalls, in a small group of healthy adults undergoing a high-FAV diet intervention [42]. The correlation coefficients between FAV intake and 24 h hippuric acid excretion, however, seemed to be higher in children than in adolescents or healthy adults [43]. These findings strengthen the rationale for using urinary hippuric acid excretion as a nutritional marker mainly in the pediatric population [38]. In adult subjects, in fact, the inconsistency between food intake and related metabolite excretion that typically emerges suggests that non-nutritional factors as determinants of hippuric acid excretion are involved [44].

Therefore, in adult subjects, the 24 h urinary hippuric acid excretion is not recommended for the precise assessment of the levels of FAV intake but can simply be used for monitoring adherence to dietary interventions [45] or for providing a raw estimation of the habitual FAV consumption in the presence of diseases that have a strong association with dietary habits, such as kidney stone disease [46]. In this condition, a low FAV intake is associated with an increased risk of renal colic [47,48]. Conversely, a high-FAV diet intervention can reduce the risk of stone recurrence [49]. Therefore, urinary hippuric acid excretion may be considered a marker of the risk of kidney stone recurrence [50].

Despite its limitations as a nutritional marker, urinary hippuric acid may in any case be associated with physiological parameters and clinical outcomes of interest in adult subjects. Urinary hippuric acid excretion levels can in fact predict the serum lipid profile in adolescence [51] and insulin sensitivity [52] in adulthood. Furthermore, in obese subjects, the levels of hippuric acid in the blood are associated with an obesity phenotype [53] and visceral fat mass [54], suggesting that this compound may also be used as a marker of metabolic health.

## 3. Hippuric Acid Metabolism in Aging and Age-Related Conditions

### 3.1. The Physiology of Hippuric Acid in Aging

In aging, the urinary 24 h hippuric acid excretion tends to increase and reaches its maximum after the age of 55 [50]. The reasons underlying this phenomenon are not completely understood and do not seem to be related simply to dietary habits. In a large study conducted on kidney stone formers of different ages, the FAV intake was significantly increased in subjects older than 55 with respect to young adults and adolescents and was significantly correlated with hippuric acid excretion [50]. However, the FAV intake of this population was, in absolute terms, well below the recommended threshold. Studies specifically conducted in aging populations from Western and low-income countries in fact uncovered that the FAV intake was inadequate in the majority of the participants [55,56].

Urinary hippuric acid excretion increases after a high-polyphenol diet challenge in older as well as in younger populations, maintaining its validity as a marker of the nutritional intake of FAV, at least in experimental conditions [57,58]. Interestingly, in older subjects, the excretion of other terminal products of polyphenol metabolism, such as vanillic acid, does not increase after a targeted high-polyphenol dietary intervention [58].

A critical step of hippuric acid synthesis is the bioavailability of glycine for conjugation with benzoic acid in the liver, as highlighted in Figure 1. Metabolic studies conducted in mouse models suggest that this pathway is unaffected by aging, contributing to explain why hippuric acid synthesis is maintained also in older age [31]. However, under stressful conditions such as after major surgery, the bioavailability of glycine in older individuals may decrease, leading to a transient condition of reduced hippuric acid synthesis [59].

Furthermore, hippuric acid clearance strongly depends on the renal function and the capacity of active secretion by OATs. Aging, even with a healthy active pattern, is always associated with a certain degree of decline in the glomerular filtration rate (GFR) [60,61]. This phenomenon is emphasized in subjects whose situation is characterized by frailty and multimorbidity, even in the absence of clear signs of chronic kidney disease (CKD) [62,63]. Furthermore, CKD itself is often underdiagnosed in older individuals because the equations for GFR estimation do not adequately account for the age-related reduction of creatinine release from skeletal muscle cells due to muscle wasting and sarcopenia [62,63].

This scenario may help to explain why a tendency towards a mild decrease in the 24 h urinary hippuric acid excretion can be observed in oldest old subjects with respect to those in the 55–70-year age group [50]. Furthermore, the hippuric acid levels in the plasma increase in older subjects, even when no clear signs of kidney disease are present [64,65]. In the Baltimore Longitudinal Study of Aging, the plasma hippuric acid levels measured in 616 adults between the ages of 38 and 94 years were positively correlated with the estimated GFR [65]. Interestingly, in a study investigating the associations between the Mediterranean diet score, a parameter strongly correlated with FAV intake, and the plasma levels of gut microbial metabolites, including hippuric acid, the correlation between plasma hippuric acid levels and renal function masked the well-known association between diet and hippuric acid metabolism [66]. Therefore, the complexity of the interaction between dietary patterns in older individuals, metabolic pathways leading to hippuric acid synthesis, and age-related decline in renal function possibly affecting hippuric acid clearance should be carefully assessed when the plasma or urine hippuric acid levels are being investigated.

### 3.2. The Role of Chronic Kidney Disease

The retention of hippuric acid in the plasma is particularly pronounced in the context of advanced CKD because the capacity of tubular secretion through OATs is impaired in that condition [67]. Pre-clinical studies also suggest that hippuric acid retention in the human body may have toxic effects, especially on the brain, kidneys and endothelium (Figure 1) [67].

In fact, hippuric acid is one of the uremic toxins responsible for the uremic syndrome associated with advanced CKD [67]. The plasma and cerebrospinal fluid levels of hippuric acid are associated with a decline in cognitive performance in neuropsychological tests [68]. However, experimental evidence suggests that hippuric acid does not directly impair the brain function but inhibits the function of OATs at the blood–brain barrier level, favoring the retention in the brain tissue of other toxins with more direct neurotoxic actions, such as indoxyl sulfate or indole acetate [21]. Additionally, hippuric acid retention in patients with advanced CKD has been associated with atherogenesis through the disruption of the endothelial function [23,24] and renal fibrosis [22].

The clinical relevance of these mechanisms, however, has been recently questioned. In a cohort of 230 Taiwanese patients with advanced CKD undergoing maintenance hemodialysis, the serum hippuric acid levels were not related to cognitive performance, despite the positive blood–brain barrier penetration ability of the compound [69]. Furthermore, the abrupt decrease in serum hippuric acid levels after kidney transplantation was not associated with significant variations in cognitive performance after three months [70].

Increased plasma levels and decreased urine levels of hippuric acid are in any case associated with an increased risk of progression of CKD of diabetic etiology according to a recent study conducted on 41 patients subjected to an untargeted metabolomic analysis of plasma samples [71]. Even moderate CKD is associated with alterations in hippuric acid clearance, with increased plasma levels and decreased urinary excretion; these changes should therefore be considered as early clinical markers of kidney disease [72].

Overall, these studies suggest that the presence of CKD should always be evaluated when hippuric acid levels in the serum and urine are being investigated.

### 3.3. The Role of Age-Related Gut Microbiota Changes

Since hippuric acid synthesis is the result of an interaction between food bioactive compounds and the gut microbiota, age-related changes in gut microbiota composition and functionality may have important consequences for hippuric acid metabolism. However, this issue has not been specifically studied to date.

In older subjects, the gut microbiota loses its stability of composition and resilience to external perturbations and is generally characterized by an increased representation of opportunistic pathogens, including Gram-negative bacteria of the *Enterobacteriaceae* family, at the expense of bacterial taxa with purported health-promoting activity, such as *Bifidobacteria, Akkermansia, Faecalibacterium* and other bacteria able to synthetize short-chain fatty acids (SCFAs) [73,74,75,76,77]. Centenarians who reach extreme ages in relatively good health generally show less pronounced changes, with the maintenance of some core taxa capable of modulating age-related chronic inflammation, gut intestinal permeability and the anabolic–catabolic balance [78,79]. Conversely, aging subjects who show signs of frailty and multimorbidity and who exhibit a poor physical and cognitive performance have generally higher levels of gut microbiota dysbiosis, i.e., an imbalance between pathobionts and symbionts [80,81]. The most pronounced degrees of dysbiosis are generally observed in older patients residing in nursing homes [82] or hospitalized for acute illness [83].

A study conducted in patients with Crohn’s disease, a gastrointestinal condition associated with marked levels of gut microbiota dysbiosis, has shown that dysbiosis reduces the capacity of the host to synthetize hippuric acid even after the administration of a sodium benzoate load as a dietary supplement [84]. Furthermore, in a group of 1529 females belonging to the TwinsUK Cohort, the plasma levels of hippuric acid were inversely associated with the Shannon Index, a measure of gut microbiome diversity [85]. Subjects with reduced hippuric acid levels were also more likely to develop metabolic syndrome and had a reduced dietary intake of FAV [85].

These findings suggest that dysbiosis may have important consequences on the metabolic pathways shown in Figure 1 and could be associated with reduced hippuric acid synthetic capacity, even when the dietary intake of FAV is high. Therefore, the presence of age-related dysbiosis should be carefully evaluated when the hippuric acid levels in plasma or urine are being assessed.

## 4. Hippuric Acid in Physical Frailty and Sarcopenia

Epidemiological studies have underlined that, in older subjects, a high dietary FAV intake is protective against frailty and its detrimental consequences [86], especially when the amounts of intake recommended by nutrition societies are met [87,88]. More specifically, high FAV intake was found to be protective against the onset of physical frailty and sarcopenia, the age-related loss of muscle mass and function associated with adverse outcomes in older subjects [89,90,91]. This association was detected in populations from different geographical regions and with different dietary patterns [86,87,88,89,90,91].

However, recent evidence suggests that this relationship may be gender-specific and that a high FAV intake is clearly protective against sarcopenia only in females [92]. Furthermore, the quality, variety and diversity of plant-based foods habitually consumed by older persons also seems to be related to the risk of developing frailty and sarcopenia, and whole grains, vegetables, nuts and legumes are considered healthier choices that are associated with the lowest risk [93,94]. In one study, the FAV variety in habitual diets was not associated with frailty or sarcopenia, but it did predict the mortality of older adults [95]. Overall, this evidence supports the recommendation of increasing the FAV intake to prevent physical frailty and sarcopenia, although most experts are convinced that more high-quality research in this field is needed [96,97,98].

Mounting evidence also suggests that gut microbiota dysbiosis is pathophysiologically involved in the onset and progression of physical frailty and sarcopenia [99,100]. The human studies supporting this hypothesis, however, were not designed to assess the hippuric acid metabolism [101].

In this scenario, measuring the urinary hippuric acid excretion may still provide important information for estimating the risk of frailty and sarcopenia [3]. Data from the Invecchiare in Chianti (InCHIANTI) Study suggest that the urinary excretion of total polyphenols is inversely associated with the phenotypical aspects of physical frailty, including exhaustion and slow walking speed [102]. The key findings of studies that specifically investigated the relationship between frailty/sarcopenia and hippuric acid metabolism in older subjects are summarized in Table 1 [103,104,105,106,107].

Overall, these studies support the hypothesis that low plasma levels of hippuric acid are associated with detrimental consequences for the physiology of older subjects, namely, muscle wasting [103], impaired muscle metabolism [106], low muscle mass [105] and frailty phenotype [104] (Table 1).

One study, instead, found results apparently in opposition to those of the others, as it uncovered that urinary hippuric acid excretion was negatively associated with the Short Physical Performance Battery (SPPB) score [107] (Table 1). According to that study, the patients with physical frailty and sarcopenia exhibited increased plasma and urine levels of hippuric acid, a profile that was shown mainly in patients with renal failure.

However, the high degree of heterogeneity of designs, sample sizes, criteria of enrollment and methods of frailty assessment is an important factor to consider when the findings of the studies listed in Table 1 are examined. In one study [106], frailty, which was considered simply in terms of alterations of glucose metabolism, was not formally assessed according to standard criteria. In another study [103], the participants were classified as “at risk for sarcopenia”, but a comprehensive evaluation of their muscle mass and function was not conducted. Given these considerations, no conclusive evidence regarding the possible role of hippuric acid as a marker of frailty, physical frailty or sarcopenia was presented by any of these studies.

Two further studies were conducted on a selected population of patients with CKD undergoing hemodialysis. The results suggested that the levels of plasma hippuric acid were negatively associated with handgrip strength, but the decline in physical performance associated with the disease was not completely explained by the retention of uremic toxins [108]. Furthermore, other uremic toxins, especially indoxyl sulfate, and not hippuric acid, were associated with skeletal muscle toxicity and sarcopenia in these patients [109].

## 5. Hippuric Acid and Cognition in Older Adults

A high FAV intake in older age is protective against cognitive decline and the onset of dementia [110,111]. This effect, which is mediated by several nutrients and bioactive compounds present in foods of vegetal origin, including polyphenols, cannot be considered the effect of a single class of molecules [112]. This relationship depends on the overall dietary pattern and not on the ingestion of a single food or a single class of foods [113]. In particular, the Mediterranean and Dietary Approaches to Stop Hypertension (DASH) style diets seem to be the best dietary patterns associated with the prevention of or the delay in cognitive decline according to nutritional epidemiology studies [113,114].

The gut microbiota also plays an important role in modulating the age-related cognitive decline [115] through multiple mechanisms that together represent the so-called microbiota–gut–brain axis [116]. The gut microbial metabolism of food bioactives, including polyphenols, may determine the synthesis of neuroprotective compounds, which represent a key mechanism involved in the microbiota–gut–brain axis [117]. Despite the potential importance of these mechanisms, comprehensive studies assessing the relationships between gut microbiota metabolic functionality and cognitive performance in human beings are still lacking [117].

In this context, high levels of urinary hippuric acid excretion may be considered inversely associated with the risk of developing age-related cognitive disorders. Interestingly, in a metabolomics study conducted on 20 patients with Alzheimer’s disease, 10 patients with mild cognitive impairment (MCI) and 29 controls, hippuric acid excretion was significantly reduced only in the MCI group, and no differences were detected when the results from the patients with dementia and the controls were compared [118]. An increase in plasma and urinary hippuric acid was also observed in intervention studies testing the cognitive effects of administering food supplements of vegetal origin, such as blueberry derivatives [119,120,121] or *Xanthoceras sorbifolium* bunge husks [122]. These studies did not, however, report any measurable effects of the nutritional intervention on cognitive performance. There is only one study that reported that an increase in plasma hippuric acid levels after the administration of a blueberry supplement was found to be associated with an improved performance on the California Verbal Learning Test in a group of 38 healthy older adults [121].

However, the relationship between hippuric acid metabolism and cognition should not be considered only from a nutritional perspective. According to the few studies conducted in human beings with dementia to date, the gut microbiota composition and function is largely disrupted in this condition [123,124,125,126,127,128,129,130], so it can reasonably be assumed that hippuric acid metabolism is markedly affected, considering the pathways leading to hippuric acid synthesis shown in Figure 1. Unfortunately, no studies have specifically investigated the plasma and urinary hippuric acid levels in patients with gut microbiota dysbiosis associated with dementia. However, in a study comparing the gut microbial metabolites in 56 patients with Parkinson’s disease, another neurodegenerative disease frequently associated with dementia, and in 43 age- and sex-matched healthy controls, plasma hippuric acid was positively associated with the disease but not with cognitive performance [131].

## 6. Hippuric Acid and Other Age-Related Chronic Conditions

Experimental studies suggest that the plasma hippuric acid levels are associated with the dysfunction of endothelial cells and involved in the pathogenesis of atherosclerosis and other major cardiovascular diseases. In patients with CKD, high plasma hippuric acid concentrations were associated with an increased carotid atherosclerotic plaque burden [66] and left ventricular hypertrophy [132]. In a group of patients with advanced peripheral atherosclerosis undergoing major vascular surgery, the preoperative plasma levels of hippuric acid were significantly associated with major cardiovascular events in the postoperative period [133]. However, in two distinct studies, the plasma hippuric acid levels exhibited a positive correlation with the ankle brachial index (ABI), an indicator inversely associated with arterial stiffness [133,134]. These findings are apparently puzzling and suggest that in patients with no impairment of kidney function, the hippuric acid levels may represent a marker of preserved arterial elasticity rather than of arterial stiffness. In fact, in patients with no CKD, the plasma hippuric acid levels reflect a good FAV intake and preserved gut microbiota composition and metabolic functionality, which are generally protective against the onset of peripheral artery diseases and other cardiovascular illnesses [135,136]. Conversely, in subjects with CKD, hippuric acid retention may represent a marker of CKD macroangiopathy.

Recent studies also suggested that urinary hippuric acid may represent a marker of colorectal and urogenital cancer. Namely, urinary metabolomics studies showed that reduced levels of urinary hippuric acid were able to discriminate in a significant manner subjects with colorectal cancer from adult subjects with no cancer diagnosis [137,138]. The levels of hippuric acid in biopsies of renal tissues with renal cell carcinoma were significantly reduced with respect to those in renal tissue samples of control subjects [139]. Furthermore, a comprehensive urinary metabolomic analysis of urine samples from patients with bladder cancer and controls revealed that hippuric acid excretion was significantly lower in the patients with the disease and before the surgical resection of the tumor [140,141].

Finally, studies conducted in animal models of rheumatoid arthritis suggest that the urinary levels of hippuric acid are inversely correlated with disease activity and markers of inflammation [142].

These studies do not, however, allow us to draw definitive conclusions on the role of hippuric acid as a biomarker of age-related diseases, but they do highlight the complexity of hippuric acid synthesis, metabolism and clearance in health and disease and indicate future directions of research.

## 7. Conclusions and Perspectives

**Figure 2:**
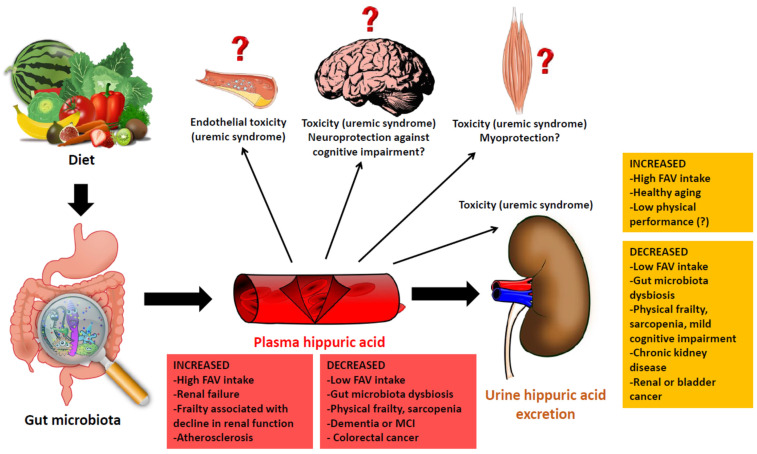
An overview of hippuric acid as a crossroads mediator between diet, microbiota, aging and organ function, with possible factors associated with its higher or lower plasma and urine levels.

The results of laboratory analyses of plasma and urinary levels of hippuric acid are influenced by multiple pathophysiological processes, including aging, age-related conditions, diet, gut microbiota composition and renal function (Figure 2). Therefore, an exhaustive evaluation of hippuric acid metabolism in human beings can provide a wealth of information on several mechanisms involved in aging. However, for the time being, the multiple factors that can influence hippuric acid synthesis, blood metabolism and urinary excretion in older individuals preclude it from being an ideal candidate for the role of biomarker of the aging trajectory.

In fact, an increase in the plasma levels of hippuric acid may be either the result of an increased FAV intake with maintained gut microbiota biodiversity or the consequence of CKD and other chronic illnesses, including dementia (Figure 2). Conversely, low plasma and urine levels of hippuric acid may simply underline an insufficient consumption of FAV in the diet as well as severe conditions associated with gut microbiota dysbiosis, including physical frailty, sarcopenia and mild cognitive impairment (Figure 2).

Unfortunately, the studies conducted to date in which the hippuric acid levels in the plasma or urine were assessed did not consider the complexity of multiple factors potentially influencing its synthesis and clearance. Instead, hippuric acid was mainly studied in relation to only one of the multiple conditions listed in Figure 2. This is perhaps the main limitation of the existing literature, especially with regard to studies focusing on older subjects and geriatric conditions. In this context, interpreting the clinical and pathophysiological significance of hippuric acid levels in the plasma and urine may result particularly challenging. Furthermore, the precise effects of hippuric acid on different organs and systems are still poorly understood. In some cases, the results of different studies seem contradictory, with some evidence suggesting that hippuric acid may exert positive physiological effects (for example, myoprotection), while other data, for the most part linked to studies conducted in patients with CKD, indicate that it may exercise a toxic effect.

**Figure 3:**
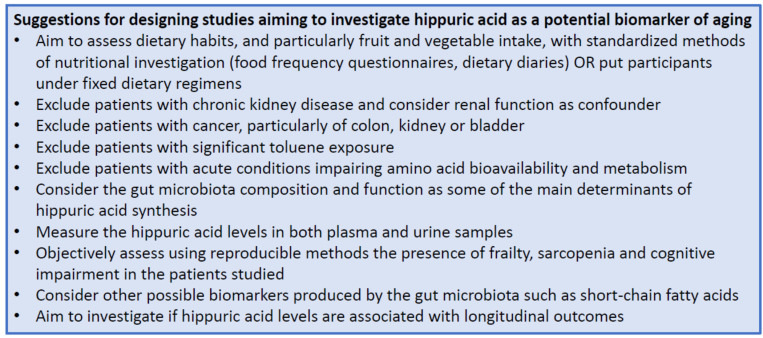
Expert opinion box with suggestions for designing studies assessing hippuric acid as a potential biomarker of aging.

Future studies will be able to contribute to disentangling these apparent contradictions and explaining the multiple confounding factors that contribute to hippuric acid synthesis, metabolism and clearance, especially with regard to aging. Some practical suggestions for designing and conducting future studies assessing the possible role of hippuric acid as a biomarker of aging trajectories are outlined in Figure 3.

In conclusion, recently published studies in the literature do not fully support the use of the plasma or urine levels of hippuric acid as a biomarker of the aging pattern. Hippuric acid synthesis and clearance are in fact influenced by multiple environmental exposures, physiological processes, chronic conditions and diseases that seem to impede efforts to interpret the laboratory results. Hippuric acid is nevertheless at the crossroads between diet, microbiota, physiological and pathological mechanisms of aging, and the study of its complex metabolism will contribute to illuminating and disentangling the complexity of the relationship between nutrition, gut microbiota and frailty linked to the trajectories of aging.

## Figures and Tables

**Table 1:** Overview of the key findings of studies that investigated hippuric acid in the plasma and urine as a marker of muscle wasting, sarcopenia or frailty in older individuals.

Author, Year (Ref)	Country	Study Design	Sample Size and Characteristics	Age	Method of Frailty Assessment	Key Findings
Saoi et al., 2019 [103]	Canada	Intervention (two weeks of inactivity with <1000 steps per day)	17 overweight pre-diabetic older adults at risk for sarcopenia	69 ± 17	Not described	Physical inactivity was associated with indirect signs of muscle wasting and reduced plasma levels of uremic toxins, including HA. Resuming normal physical activity was not associated with recovery of baseline HA levels.
Brunelli et al., 2021 [104]	Italy	Longitudinal population-based	Profiling cohort: 65 fit and 65 frail Validation cohort: 124 fit, 59 pre-frail and 81 frail	Range 76–78 y.o. for all cohorts	Frailty Index based on 32 health variables or deficits	HA was the only marker, detected by an untargeted metabolomic approach, significantly lower in the plasma of the frail with respect to that of fit subjects and linearly associated with the FI. HA levels predicted incident frailty.
Kameda et al., 2021 [105]	Japan	Cross-sectional	19 older community dwellers	86 ± 7	Skeletal Muscle Index measured by bioimpedance analysis; gait speed on 10 m straight walkway; grip strength	HA was among 22 plasma markers of sarcopenia with significantly lower levels in patients with low SMI detected by bioimpedance analysis, but was not associated with frailty.
Harmsen et al., 2022 [106]	Netherlands	Cross-sectional	12 older metabolically compromised men 12 young fit adults	65 ± 9 (older) 22 ± 2 (young)	Altered glucose metabolism	HA was significantly reduced in the plasma of older men and associated with a marker of altered skeletal muscle metabolism. The amplitude of plasma HA variations over the day was reduced in older men.
Douzi et al., 2022 [107]	Finland	RCT	33 patients undergoing rehabilitation after hip fracture surgery	80 ± 8	Fried phenotypical criteria of frailty	Urinary HA was significantly increased in patients with low physical performance, measured by the SPPB score, and in patients who died during hospital stay.

HA = hippuric acid; FI = Frailty Index; SMI = Skeletal Muscle Index; SPPB = Short Physical Performance Battery; RCT = Randomized Controlled Trial.

## Data Availability

Not applicable.
